# AN AGE-FRIENDLY COURSE ADOPTING THE 4M GERIATRIC MODEL IN A PRIMARY CARE NURSE PRACTITIONER PROGRAM

**DOI:** 10.1093/geroni/igac059.2741

**Published:** 2022-12-20

**Authors:** Ophelia Empleo-Frazier, Ami Marshall, Margaret Doyle, Andrea Rink, Noelle Gallant, Barry Wu, Richard Marottoli

**Affiliations:** Yale University School of Nursing, Orange, Connecticut, United States; Yale University School of Nursing, Orange, Connecticut, United States; Yale University, New Haven, Connecticut, United States; Yale University School of Medicine, New Haven, Connecticut, United States; Yale New Haven Health System, New Haven, Connecticut, United States; Yale University School of Medicine, New Haven, Connecticut, United States; Yale University, New Haven, Connecticut, United States

## Abstract

Training nurse practitioners to assume an expanded role in management of geriatric patients is key to addressing the care needs of a growing older adult population. To that end, the Yale School of Nursing revised geriatric coursework required of family nurse practitioner (FNP) and adult geriatric nurse practitioners (AGNP) to ensure the curriculum was sufficiently in-depth, engaging, and inclusive. Revisions included a new course, “Advanced Primary Care of the Older Adult” which focuses on the role of the nurse practitioner in the assessment, diagnosis, and management of primary geriatric syndromes. The 4M model (Medications, Mentation, Mobility, and what Matters most) provided the framework. Additionally, faculty added expert speakers, interactive dementia animated videos, book and movie discussions and a geriatric telehealth simulation component. Content was guided by training needs data collected from prior nurse practitioner students. At the end of each course, students were surveyed to assess attitudes towards geriatric care and satisfaction with the course. We compared survey responses pre (academic year 2019–2020, Nf18, response rate=53%) and post (academic years 2020–2021 and 2021–2022, Nf28, response rate=44%) rollout. In multivariate regression analyses adjusted for differences in age, gender, race, program (AGNP and FNP) and previous geriatric experience, we found significant improvement in measures of both confidence in providing geriatric care and satisfaction with course content. Analysis of qualitative data confirmed student satisfaction with the new course. Future work will include expansion and more rigorous evaluation of the program, including measures of student knowledge of best practices and increasing survey participation rates.

